# Using Multiple Sources of Data to Assess the Prevalence of Diabetes at the Subcounty Level, Duval County, Florida, 2007

**Published:** 2010-08-15

**Authors:** William C. Livingood, Luminita Razaila, Elena Reuter, Rebecca Filipowicz, Ryan C. Butterfield, Katryne Lukens-Bull, Linda Edwards, Carlos Palacio, David L. Wood

**Affiliations:** Duval County Health Department and University of Florida College of Medicine, Jacksonville; Duval County Health Department, Jacksonville, Florida; Duval County Health Department, Jacksonville, Florida; Duval County Health Department, Jacksonville, Florida; Duval County Health Department, Jacksonville, Florida; University of Florida College of Medicine, Jacksonville, Jacksonville, Florida; University of Florida College of Medicine, Jacksonville, Jacksonville, Florida; University of Florida College of Medicine, Jacksonville, Jacksonville, Florida; University of Florida College of Medicine, Jacksonville, Jacksonville, Florida

## Abstract

**Introduction:**

Diabetes rates continue to grow in the United States. Effectively addressing the epidemic requires better understanding of the distribution of disease and the geographic clustering of factors that influence it. Variations in the prevalence of diabetes at the local level are largely unreported, making understanding the disparities associated with the disease more difficult. Diabetes death rates during the past 15 years in Duval County, Florida, have been disproportionately high compared with the rest of the state.

**Methods:**

We analyzed multiple sources of secondary data related to diabetes illness and death in Duval County, including data on hospital discharge, emergency department (ED) use, and vital statistics. We accessed diabetes and diabetes-related ED use and hospitalization and death data by using codes from the International Classification of Diseases versions 9 and 10. We analyzed data from the Behavioral Risk Factor Surveillance System survey for Duval County and adapted Centers for Disease Control and Prevention weighting formulas for subcounty analysis. We used relative risk-type disease ratios and geographic information systems mapping to analyze data.

**Results:**

The urban, mostly minority, low-socioeconomic area of Duval County had twice the rate of diabetes-related illness and death as other areas of the county, and the inner-city, poor area of the county had almost 3 times the rate of hospitalization and ED use for diabetes and diabetes-related conditions compared with the other areas of the county.

**Conclusion:**

Our analyses show that diabetes-related disparities affect not only people and their families but also the community that absorbs the costs associated with the disproportionate health care use that results from these disparities. Analyzing data at the subcounty level has implications for health care planning and public health policy development at the local level.

## Introduction

Diabetes is recognized as a growing national and international epidemic as prevalence rates for other major chronic diseases such as stroke and heart disease have decreased (1,2). The challenges of addressing the epidemic are exacerbated by the disparities in the prevalence of the disease. These disparities are complicated by quality-of-care issues and socioeconomic determinants (3-7), which may include local geographically clustered factors such as availability and access to health care, education and employment opportunity, and social capital and social cohesion. Furthermore, variations in the prevalence of diabetes at the local level are largely unreported, making understanding the disparities associated with the disease more difficult.

**Figure 1.**
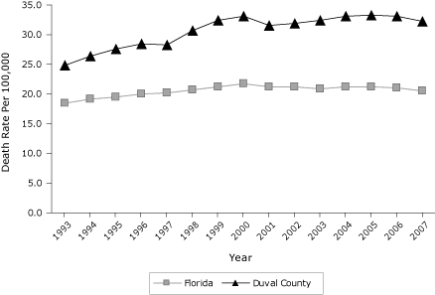
Yearly trend in age-adjusted diabetes 3-year death rate per 100,000. Three-year rate is calculated by summing the 3 years of deaths and dividing by 3 to obtain the annual average of events, followed by calculating the age-specific rates for each year. Data source: Florida Department of Health, Bureau of Vital Statistics, 1998-2008.

Duval County is a consolidated city/county government located on the northeast coast of Florida. It is a large (more than 840 square miles) and diverse area that has a population of more than 900,000 (8). The city/county contains areas that reflect urban, suburban, and rural areas. In 2007, Duval County, which encompasses Jacksonville, had an age-adjusted diabetes death rate of 32 per 100,000, compared with the 10 other largest counties in Florida (range, 14-29; median = 20) (9). In 2007, the total hospitalization costs for adult diabetes-related treatment in Duval County exceeded $714,000,000, and the cost for emergency department (ED) visits due to diabetes-related treatment was more than $57,000,000 (10). The growing disparity in diabetes deaths between Duval County and the state of Florida as a whole has been an alarming trend during the past 15 years (Figure 1).

**Table:** 

**Year**	**Death Rate Per 100,000 Population**	
	**Florida**	**Duval County**
1993	18.5	24.8
1994	19.1	26.4
1995	19.6	27.5
1996	20.0	28.4
1997	20.1	28.3
1998	20.7	30.8
1999	21.3	32.4
2000	21.7	33.0
2001	21.3	31.5
2002	21.1	32.0
2003	20.8	32.5
2004	21.2	33.1
2005	21.2	33.3
2006	21.1	33.2
2007	20.6	32.1

We used several methods to study the local prevalence of diabetes, including the use of administrative data for the number of hospital and physician visits for diabetes (11,12) and Behavioral Risk Factor Surveillance System (BRFSS) survey self-reported data (13). Both methods rely on health system diagnosis, either documented through administrative records or communicated to patients. We used a method of weighting prevalence rates to adjust for undiagnosed cases (14). Diabetes death rates and various measures of diabetes prevalence capture different forms of observable characteristics or effects of the disease. We assessed the comparable sensitivity of these measures, particularly as the measures relate to geographic distribution of ethnicity and social determinants, and analyzed diabetes-related disparities at the local level by using different sources of data to provide implications for public health and health care policy.

## Methods

We used a secondary data analysis research design that included multiple sources to assess the prevalence and effect of diabetes in Duval County. Data sources were ED and hospital discharge data for the year 2007 reported to the Florida Agency for Health Care Administration (AHCA), vital statistics data for the year 2007 reported to the Florida Department of Health, 2007 BRFSS data collected by the Florida Department of Health, population data collected by the US Census Bureau and census estimates generated by the Florida Office of Economic and Demographic Research and Nielsen Claritas, and previously created geographically defined areas, identified as *health zones*.

### Data sources and management

**ED and hospital discharge data.** Hospitals in Florida are required to report ED and hospitalization data quarterly to the AHCA, using a standardized format based on codes from the International Classification of Diseases (ICD) version 9. The most current complete data file available at the time of the analysis was 2007. Using hospital discharge data for all people aged 18 years or older, we identified the ICD-9 codes for diabetes (all diseases and conditions coded as 250) as the primary cause of hospitalization or ED use. Then, we counted diabetes-related cases as admissions for which the primary diagnosis was diabetes or for which diabetes was coded as a contributing condition. Finally, we calculated the rates by dividing the frequencies for diabetes or diabetes-related cases by the population and multiplying by 100,000.

**Vital statistics death file data.** First, we used ICD-10 codes to identify diabetes deaths from the primary cause of death and diabetes-related deaths from all contributing causes of death in addition to the primary cause of death, as recorded on the death certificate. Next, we calculated rates by dividing the number of cases in people aged 18 years or older by the population in each geographic area or demographic group and multiplying by 100,000.

**BRFSS data.** The Florida Department of Health conducted the 2007 BRFSS survey in the state of Florida. The Duval County Health Department obtained a larger sample from the county population so that we could conduct analyses at the subcounty level. The larger sample was purchased through the Florida Department of Health using noncategorical discretionary funds available to the county health department. Approximately 1,800 residents aged 18 years or older responded to the BRFSS in Duval County. The responses were weighted by using BRFSS weighting methods that account not only for the sampling plan of the telephone survey but also for the distribution of demographic groups within the county (see Appendix for description of weighting). The variable of interest from the BRFSS data file was DIABETE2, which contained the answer to the survey question, "Have you ever been told by a doctor that you have diabetes?" We counted the numbers of affirmative responses on both the raw data file and weighted data file and used them to calculate the weighted and unweighted BRFSS prevalence rates for diabetes, using SPSS software (SPSS, Inc, Chicago, Illinois). We calculated undiagnosed cases as a proportion of diagnosed cases (14).

**Population data.** We obtained the population estimates used for county-level rates from the Florida Office of Economic and Demographic Research via the Florida Department of Health, Office of Health Statistics and Assessment (CHARTS) (15). Population estimates for the subcounty level rates were obtained from Nielsen Claritas, a demographic data vendor that provided 2000 census–based demographic projections by zip code. The Florida Office of Economic and Demographic Research provides official state estimates for the county, but Nielsen Claritas was needed for the subcounty estimates.

**Figure 2.**
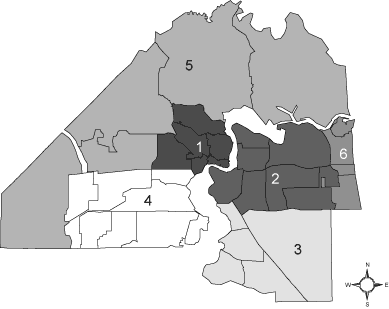
Health zones, Duval County, Florida. Prepared by the Duval County Health Department, Institute for Health, Policy, and Evaluation Research, August 2008.

**Subcounty divisions.** Because the zip code areas of Duval County were statistically unreliable for many health issues, we used multi–zip code health zones (Figure 2) that were created by the Duval County Health Department Institute for Health, Policy, and Evaluation Research to provide reliable and consistent data for subcounty analysis (16). Data generated on the basis of health zones also overcome Health Insurance Portability and Accountability Act (HIPAA) issues concerning protection of personal identifiers associated with geographic areas with small populations. The private and public health and social services sectors of the county use these health zones extensively for community assessment and planning. The health zones have different demographic characteristics. For example, health zone 1 is more than 80% African American, whereas health zones 3 and 6 are less than 20% African American. Health zone 1 has many health disparities compared with the other health zones (16).

### Analysis

Comparison of rates from the different data sources involved several steps: calculation of rates using a standard format; comparison of rates for each of the subcounty zones to the overall county rate, using graphic and mapping analytic techniques; calculation of the disease ratios (relative risk and prevalence ratio) for the urban core zone (health zone 1) compared with the rest of the county; and calculation of odds ratios for comparison of each health zone to one another. Disease ratios such as relative risk (typically associated with incidence) and prevalence ratios use the same formulas for calculation. The relative risk and corresponding confidence interval calculations were computed by using Epi Info (Centers for Disease Control and Prevention, Atlanta, Georgia).

**GIS mapping.** We used geographical information systems (GIS) mapping to interpret and visualize patterns of diabetes illness and death and hospitalization and ED data across Duval County health zones. We used ArcMap (Environmental Systems Research Institute, Inc, Redlands, California) for spatial analysis. Specifically, we developed thematic maps using percentages and rates of disease by health zone.

**Disease ratios (relative risk and prevalence ratio).** We compared illness and death rates by dividing the rate for health zone 1 by the rate for the rest of Duval County for each diabetes measure. The prevalence rate was based on the BRFSS weighted sample. We constructed a graph to compare the diabetes rates in health zone 1 (the urban core) with the rest of the county in the rank order of diabetes measures derived from the rates per 100,000. We then calculated confidence intervals for the disease ratios (prevalence ratios and relative risks), comparing health zone 1 against the other health zones for each diabetes measure.

## Results

**Figure 3.**
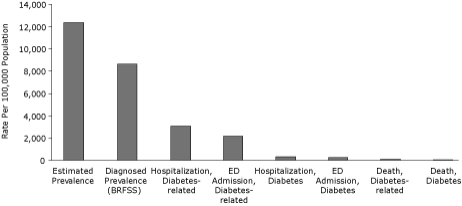
Rates for diabetes measures in Duval County, Florida, 2007. Rates are presented in descending order, on the basis of number of cases per 100,000 population. Abbreviations: BRFSS, Behavioral Risk Factor Surveillance System; ED, emergency department. Data sources: BRFSS, Duval County, 2007; Florida Agency for Health Care Administration, in-patient and ED data, 2007; Florida Department of Health, Office of Vital Statistics, 2007 death files.

Diabetes rates vary extensively in Duval County, depending on the source of data and the type of measure. Figure 3 shows the relationship of these measures in descending order, ranging from an estimated diabetes prevalence of 12,371, per 100,000 population to a death rate of 40 per 100,000.

**Table:** 

**Measure**	**Rate Per 100,000 Population**
Estimated prevalence	12,371
Diagnosed prevalence (BRFSS)	8,660
Hospitalization, Diabetes-related	3,098
ED Admission, Diabetes-related	2,173
Hospitalization, Diabetes	306
ED Admission, Diabetes	242
Death, Diabetes-related	94
Death, Diabetes	40

Overall, residents from health zone 1 are less educated and poorer than residents from the other health zones, and health zone 1 has a higher African American population than the other health zones (Table 1).The extensive local variations for these different measures are illustrated in Table 2, which shows major disparities in the county. Health zone 1, the urban core, had an age-adjusted death rate of 93.5 compared with the lowest rates in the county, health zones 2, 3, and 6 with rates of 30.5, 31.0, and 31.6, respectively. These 3 rates were lower than the county rate of 39.9 and the state rate of 34.9. The other health zones (4 and 5) had rates that were less than half of the urban core rate. The rate of age-adjusted diabetes death for adults varied dramatically by health zone. Health zone 1 had more than double the rate of health zone 5, which had the next highest rate, and more than triple the 2 lowest rates (health zones 2 and 3).

Rates for hospitalization and ED visits revealed even more profound disparities in terms of location. The hospitalization rate for the urban core (health zone 1) was 747 compared with 148 for the health zone with the lowest rate (health zone 3). The urban core hospitalization rate was more than double that of all other health zones but 1. The distribution of diabetes rates for ED use was similar in that the rate of health zone 1 far exceeded those of the other health zones. Health zone 1 had an unusually high ED visit rate (692) compared with health zone 3, which had the lowest rate (105) and had more than twice the rate of the other health zones. Health zone 1, which had a rate of self-reported diagnosed diabetes of 14,250, exceeded the county rate, but this is the only measure for which another health zone (health zone 5, rate of 15,446) exceeded the urban core (ie, health zone 1) (Table 2).

The ratios of prevalence and relative risk in health zone 1 compared with the rest of the county for each diabetes measure complemented the GIS analysis, providing markers of significance for the disparities in the county (Table 3). Significant differences between health zone 1 and the rest of the county (health zones 2-6) were established for each of the diabetes measures. The largest difference in ratios was for diabetes ED use, followed by diabetes-related ED use. The health zone 1 ratio for hospitalization for diabetes and diabetes-related illness were also high compared with the other zones. The ratios for diabetes deaths and diabetes-related deaths were also comparatively high for health zone 1. The ratio of prevalence of diabetes for health zone 1 compared with the other health zones was the lowest ratio.

## Discussion

The results of our study show that the diabetes prevalence ratios within the high-minority, low-socioeconomic area of Duval County were statistically different when compared with the other parts of the county. Understanding the effect of the disease and the distribution of that disease in the community has implications for policy development and resource allocation. The costs associated with hospitalization and ED use are much higher in the high-minority, low-socioeconomic part of the county. The cost per capita of diabetes-related hospitalizations in health zone 1 in 2007 was $2,010, which was nearly double the cost per capita for the county ($1,059). The charity cost per capita of diabetes-related hospitalizations in health zone 1 in 2007 was $1,053, which was more than double the cost per capita for the county ($465). The reasons for the acute disparities identified by this study deserve considerably more discussion than is feasible here, but they include a range of socioeconomic and health care disparities (17-29).

The results of this study provide insights about the distribution of diabetes in specific areas of the county, insights that get lost in data aggregated at the metropolitan level. An unexpected result of our study was the low rate of diagnosed cases of diabetes, which were inferred from BRFSS data. This could be due to a lack of access to prevention and primary care for people in health zone 1, resulting in poorer outcomes related to delayed care, which are reflected in the other measures such as higher rates of hospitalization and death, as previously discussed. However, it may also reflect flaws in BRFSS methods related to low participation of African Americans in the BRFSS telephone surveys, which is exacerbated by declining land-line use. Although weighting is used to compensate for underrepresentation, it may not adequately address disproportionate underrepresentation of the highest-risk patients among African Americans.

Our study has limitations that are associated with most efforts to measure disease, illness, and death. The accuracy of the data is dependent on the people observing and recording the data and may be affected by the data collection process. Another limitation is that BRFSS data use sampling frames and telephone interviews that have inherent issues with sampling bias, particularly when refusals and land-line issues are considered. However, examining multiple sources of data is beneficial because together these sources provide a more accurate picture of disease effect, similar to the concept of triangulation found with qualitative research.

Currently allocated resources may be insufficient or inappropriate to adequately deal with diabetes and its complications in the areas of highest need. Health zone 1 is the urban core, which has the lowest socioeconomic levels. Areas with the highest prevalence of diabetes contain the patients who have the fewest resources to deal effectively with the disease. This disparity may account for the disproportionate number of hospital and ED visits, which drive up the cost of health care for the poorest because of a lack of adequate preventive resources.

Our analyses revealed that diabetes disproportionately affects the geographic part of the community that has the highest minority population and the lowest socioeconomic status. The most sensitive measures of the effects of diabetes at the local level were hospitalization data and ED use, and the least sensitive measure was prevalence, determined from BRFSS data. Our analyses show that diabetes-related disparities affect not only people and their families, but also the community that absorbs the costs associated with the disproportionate health care use that results from these disparities. Analyzing data at the subcounty level has implications for health care planning and public health policy development at the local level.

## Figures and Tables

**Table 1:** Health Zone Comparisons for Selected Demographic Characteristics, Duval County, Florida, 2007^a^

**Characteristic^b^**	Health Zone 1	Health Zone 2	Health Zone 3	Health Zone 4	Health Zone 5	Health Zone 6	Duval County
Residents at or below federal poverty level	28.0	8.8	5.3	11.7	10.8	7.3	11.9
At least high-school education	63.7	87.2	92.5	82.6	75.7	89.6	82.9
Children aged <18 y at or below the federal poverty level	38.4	12.0	6.4	16.6	14.5	9.2	16.4
Average median household income, $	21,185	44,509	53,972	39,610	42,040	44,765	41,118
African American	79.2	19.8	9.3	21.4	27.7	10.8	27.8

a Data source: US Census, 2000.

b All numbers are percentages unless otherwise indicated.

**Table 2:** Diabetes Rates by Health Zone, Duval County, Florida, 2007^a^

Health Zone	Measure			
	Deaths^b^	Hospitalizations^c^	ED Visits^c^	Diagnosed With Diabetes^d^
1	93.5	747	692	14,250
2	30.5	239	191	6,310
3	31.0	148	105	5,166
4	38.8	322	236	11,861
5	43.8	401	224	15,446
6	31.6	187	155	5,132

Abreviation: ED, emergency department

a Rates are per 100,000 adult population.

b Age-adjusted. Source: Florida Department of Health, Office of Vital Statistics.

c Source: Florida Agency for Health Care Administration.

d Data obtained from the 2007 Behavioral Risk Factor Surveillance System and reflect participants who responded yes to the question, "Have you ever been told by a doctor that you have diabetes?"

**Table 3:** Ratio of Diabetes Illness and Death for Health Zone 1 vs Other Health Zones, Duval County, Florida, 2007

**Data Source**	Ratio (95% CI)
Diagnosed prevalence^a^	1.67 (1.64-1.70)
Hospitalization, diabetes-related	2.49 (2.41-2.56)
ED use, diabetes-related	3.37 (3.26-3.48)
Hospitalization, diabetes	2.94 (2.68-3.22)
ED use, diabetes	3.74 (3.38-4.13)
Death, diabetes-related	2.52 (2.12-3.00)
Death, diabetes	2.70 (2.08-3.51)

Abbreviations: CI, confidence interval; ED, emergency department.

a Data obtained from the 2007 Behavioral Risk Factor Surveillance System.Data sources: Behavioral Risk Factor Surveillance System, Duval County, 2007; Florida Agency for Health Care Administration, in-patient and emergency department data, 2007; Florida Department of Health Office of Vital Statistics, 2007 death files.

## Appendix. Method for Weighting Data

The weighting formula for the data was adapted from the Behavioral Risk Factor Surveillance System method for calculating "FINALWT" (www.cdc.gov/BRfss/technical_infodata/weighting.htm). A variable, "HZFINALWT," was created, which was the final weight assigned to each respondent. It was obtained by replacing "POSTSTRAT" with "ZONEPOSTRAT" in the formula of "FINALWT." It was calculated by multiplying 2 variables, "WT2" and "ZONEPOSTSTR." The variable "WT2" (equals STRWT × 1/NPH × NAD) is precomputed by Centers for Disease Control and Prevention and takes into account the number of adults in the respondent's household, the inverse of the number of residential telephone numbers in the respondent's household, and the differences in the basic probability of selection among strata. The variable "ZONEPOSTSTR" was created to account for zone, age, race, and sex of the respondent and is equal to the number of people in a health zone-by-age-by-race-by-sex category divided by the sum of the products of the preceding weights for the respondents in that same health zone-by-age-by-race-by-sex category (30).
